# KnowVID-19: A Knowledge-Based System to Extract Targeted COVID-19 Information from Online Medical Repositories

**DOI:** 10.3390/biom14111411

**Published:** 2024-11-06

**Authors:** Muzzamil Aziz, Ioana Popa, Amjad Zia, Andreas Fischer, Sabih Ahmed Khan, Amirreza Fazely Hamedani, Abdul R. Asif

**Affiliations:** 1 Future Networks, eScience Group, Gesellschaft für Wissenschaftliche Datenverarbeitung mbH Göttingen (GWDG), 37077 Göttingen, Germany; 2 Institute for Clinical Chemistry, University Medical Center Göttingen, George-August-University, 37073 Göttingen, Germany; 3 Hector Institute for Artificial Intelligence in Psychiatry (HITKIP), Central Institute of Mental Health (CIMH), 68159 Mannheim, Germany

**Keywords:** knowledge-based system, natural language processing, web crawling and scraping, COVID-19, long COVID, artificial intelligence, text mining

## Abstract

We present KnowVID-19, a knowledge-based system that assists medical researchers and scientists in extracting targeted information quickly and efficiently from online medical literature repositories, such as PubMed, PubMed Central, and other biomedical sources. The system utilizes various open-source machine learning tools, such as GROBID, S2ORC, and BioC to streamline the processes of data extraction and data mining. Central to the functionality of KnowVID-19 is its keyword-based text classification process, which plays a pivotal role in organizing and categorizing the extracted information. By employing machine learning techniques for keyword extraction—specifically RAKE, YAKE, and KeyBERT—KnowVID-19 systematically categorizes publication data into distinct topics and subtopics. This topic structuring enhances the system’s ability to match user queries with relevant research, improving both the accuracy and efficiency of the search results. In addition, KnowVID-19 leverages the NetworkX Python library to construct networks of the most relevant terms within publications. These networks are then visualized using Cytoscape software, providing a graphical representation of the relationships between key terms. This network visualization allows researchers to easily track emerging trends and developments related to COVID-19, long COVID, and associated topics, facilitating more informed and user-centered exploration of the scientific literature. KnowVID-19 also provides an interactive web application with an intuitive, user-centered interface. This platform supports seamless keyword searching and filtering, as well as a visual network of term associations to help users quickly identify emerging research trends. The responsive design and network visualization enables efficient navigation and access to targeted COVID-19 literature, enhancing both the user experience and the accuracy of data-driven insights.

## 1. Introduction

The COVID-19 pandemic has generated an unprecedented amount of biomedical data, with thousands of research articles, clinical trials, and sequence read archives being published daily. As of January 2024, the NCBI website alone hosts 6,735,990 sequence read archive (SRA) runs, 8,668,973 nucleotide records, 9509 clinical trials, 408,876 PubMed articles, and 654,880 PubMed Central (PMC) articles related to COVID-19 and long COVID [1,2]. This vast amount of data presents both opportunities and challenges for researchers seeking to extract valuable knowledge and insights.

Traditional text mining tools were employed to analyze this data, but they are often limited by their accuracy, scalability, and flexibility. Moreover, these tools struggle to handle unstructured data, which comprises a significant portion of the available literature. To overcome these limitations, alternative approaches such as rule-based systems, knowledge graphs, semantic analysis, ontology-based methods, and machine learning (ML)-based text mining were explored. However, a robust and adaptable approach that can efficiently extract targeted information from the vast COVID-19 literature is still lacking.

To address this challenge, we developed the KnowVID-19 system, a hybrid approach that combines traditional and ML-based text mining techniques to create a comprehensive knowledge graph of COVID-19 and long COVID medical information. Our primary objective was to design and implement an efficient and accurate ML-based text mining solution that can systematically analyze and extract relevant information from the diverse biomedical data related to COVID-19. The KnowVID-19 system aims to provide a deployable web application that enables researchers to selectively and comprehensively explore the knowledge graph, thereby enhancing the extraction of valuable knowledge from the extensive COVID-19 literature and contributing to more time-efficient biomedical research.

The KnowVID-19 system’s adaptability is a notable strength, allowing it to be easily customized for various scientific subjects with minimal effort. By leveraging artificial intelligence techniques, our system can provide a robust and adaptable tool for researchers, ultimately facilitating more efficient and accurate information extraction from online medical repositories.

## 2. Description of the Approach

**Figure 1:**
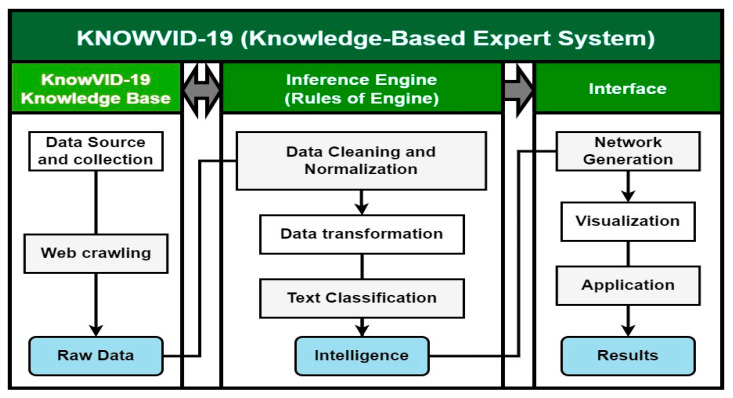
Elements and processes of the KnowVID-19 KBS.

The KnowVID-19 system is a knowledge-based expert system (KBS) that leverages artificial intelligence (AI) techniques to aid in acquiring knowledge related to COVID-19. The system consists of three components: the knowledge base, the inference engine, and the human/computer interface (Figure 1).

The knowledge base serves as a central repository, storing knowledge as ontologies. It is designed to gather raw data from diverse sources, including PubMed and PMC, using custom-designed web crawlers. The inference engine, often referred to as the cognitive core of the KBS, plays a pivotal role in analyzing and extracting relevant information from the knowledge base. It utilizes AI techniques, including machine learning and natural language processing (NLP), to draw conclusions or deduce novel information based on the knowledge base.

### 2.1. KnowVID-19—Knowledge Base

The knowledge base is the core-structured repository housing essential facts, rules, and heuristics specific to the COVID-19 domain. It is fundamental to the KnowVID-19 system, providing the foundation for intelligent decision support.

#### 2.1.1. Data Source and Collection

The KnowVID-19 uses PubMed and PMC as its primary data sources, accessing and compiling diverse COVID-19-related information in various formats such as PDF, Excel, and JSON. To collect relevant literature, we employed specific search terms and strings, including “COVID-19”, “SarsCoV-2”, and related keywords.

The search terms and strings used to collect the most relevant literature are listed below:

Full COVID-19 publication list:

- (2019-ncov OR coronavirus OR coronavirus disease 2019 OR COVID-19 OR novel coronavirus OR novel coronavirus pneumonia OR SARS-CoV-2) AND (2019 [Date—Publication]: 2022 [Date—Publication]) AND (English [Language])
- “2019 NCOV” [all fields] or “COVID-19 nucleic acid testing” [all fields] or “COVID-19 nucleic acid testing” [MeSH term] or “COVID-19 serology testing” [all fields] or “COVID-19 Serological testing [MeSH term] or “COVID-19 serotherapy” [all fields] or “COVID-19 serotherapy” [all fields] or “COVID-19 testing” [all fields] or “COVID-19 test” [all areas] COVID-19” [MeSH term] or “COVID-19 vaccine” [all fields] or “COVID-19 vaccine” [MeSH term] or “COVID-19” [all fields] or “COVID-19” [all fields] MeSH] or “NCOV” [all fields] or “SARS-CoV-2” [all fields] or “SARS-CoV-2” [MeSH term] or “Severe Acute Respiratory Syndrome Coronavirus 2” [all fields]

Specified by keyword or topic: Keyword/Topic + COVID-19 or SARS-CoV

- Topic: vaccination, drugs, non-drugs, intervention, and mental health

The initial dataset comprises publications obtained through the PubMed search engine, encompassing those featuring the specified search term or string within their publication title, abstract, or body text. The initial test dataset consisted of 4222 publications focused on the primary theme of COVID-19 vaccination, spanning the publication period from 2019 to 2022. Thereafter, the database was augmented with additional publications from PMC, resulting in a total of 24,372 publication records and 9284 publications with full-text content.

#### 2.1.2. Web Crawling

**Figure 2:**
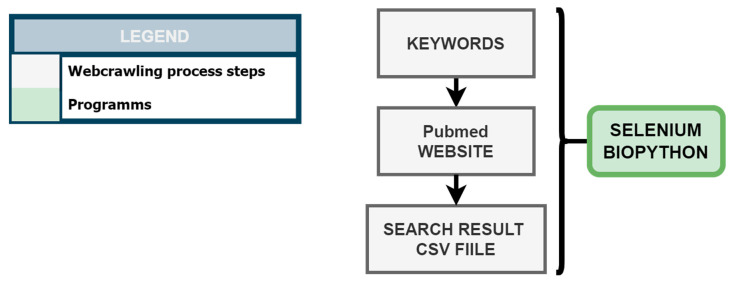
Web crawling process of COVID-19 related publications from the PubMed website.

KnowVID-19 employs a web crawling methodology, as illustrated in Figure 2, to systematically gather information from crucial biomedical online sources, specifically targeting PubMed and PMC. A Python-based web crawler was developed utilizing Selenium (https://selenium-python.readthedocs.io/, accessed on 3 November 2024) and BioPython (https://biopython.org/) libraries to extract publications from these databases based on predefined search terms and strings (as mentioned in Section 2.1.1).

The web crawling process initiates with the generation of an NLP-based search string list, which is subsequently employed to query PubMed and retrieve relevant search results. These results are subsequently stored in a CSV file format.

Performance wise, the web crawler is designed to be highly flexible and can potentially gather data from any prepeer-reviewed and peer-reviewed source, such as Google Scholar, medRxiv.org, www.biorxiv.org, and arXiv.org. It has performed smoothly during scaling attempts, demonstrating its capability to handle multiple times bigger datasets and accommodate the growing amount of COVID-19 research data. This scalability has been achieved through several strategies, as outlined below:

- The web crawler employs incremental crawling, allowing the system to detect and retrieve only newly added or updated content from previously indexed sources. This approach minimizes redundant data retrieval, which significantly reduces the processing load and enables the system to stay up-to-date more efficiently.
- The crawler incorporates an automated scheduling mechanism through a Python script that performs regular database refreshes, ensuring that new publications are integrated into KnowVID-19 without requiring manual intervention.

### 2.2. Inference Engine (Rules of Engine)

The inference engine is the cognitive core of the KnowVID-19 system, responsible for analyzing and extracting relevant information from the knowledge base. It utilizes AI techniques, including machine learning and natural language processing (NLP), to draw conclusions or deduce novel information based on the knowledge base.

#### 2.2.1. Data Cleaning and Normalization

**Figure 3:**
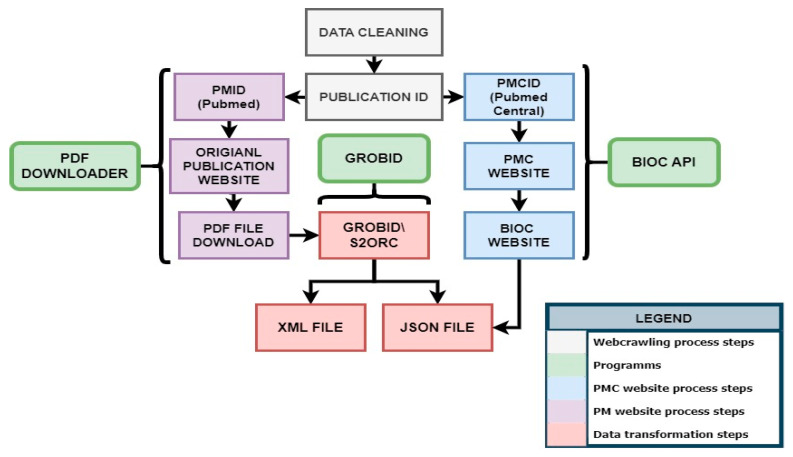
Processing of the crawled raw data.

Data cleaning and normalization are essential preprocessing steps that ensure the quality and integrity of the data [3]. The KnowVID-19 system employs various tools and Python libraries, including NumPy [4], and Pandas, to clean and normalize the data. The process involves excluding inaccurate, duplicate, corrupt, or poorly formatted entries, as illustrated in Figure 3.

#### 2.2.2. Data Transformation

**Figure 4:**
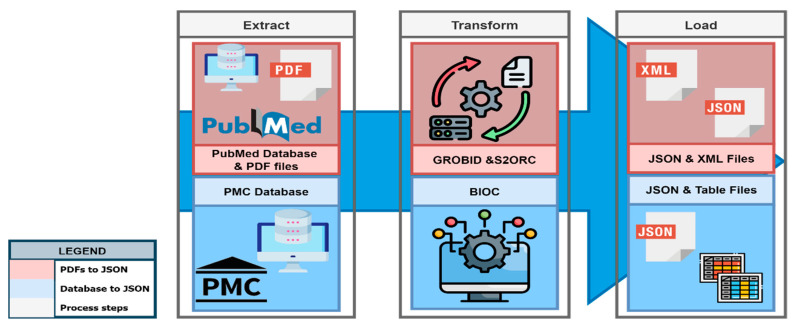
Data transformation process, from data extraction to loading the data in different file formats.

After gathering all the necessary data from several sources, the data is transformed into an appropriate format (XML, JSON, or Excel). This transformation process may involve multiple distinct or intricate steps, depending on the required modifications (see Figure 4). The transformation steps differ for publications with PubMed identifier (PMID) and PubMed Central identifier (PMCID).

For PMID publications, tools like GROBID (https://github.com/kermitt2/grobid, all web accessed on 3 November 2024) (GeneRation of Bibliographic Data—a ML library) and S2ORC (https://github.com/allenai/s2orc) [5] (Semantic Scholar Open Research Corpus) are utilized to extract and structure the information from scholarly documents; the BioC (https://bioc.sourceforge.net/) [6] API is employed to extract and structure the information from PMCID publications. The transformed data formats are easy to preprocess text and/or data, making it easier to integrate the data into diverse programs and systems. The data transformation process also involves a postprocessing step to rectify errors and address missing values [6,7]. Following the transformation, the data becomes more structured and organized, making it easier to utilize by both humans and computers.

#### 2.2.3. Text Classification

Text classification is the most crucial step in the success of KnowVID-19. It categorizes the publication data downloaded from the web crawler into topics and subtopics that are later used to correctly identify the content a KnowVID-19 user is interested in. The KnowVID-19 system employs a hybrid approach, combining manual (in the supervision of domain expert) and automatic (the use of machine learning tools) text classification.

#### 2.2.4. Text Classification Based on Manual Keywords Generation

Manual keywords generation involves creating a list of keywords that are categorized into main topics, topics, subtopics, and keywords. This list is generated by the medical researchers from the Institute for Clinical Chemistry of the University Medicine Göttingen (UMG). Each major topic is broken into multiple subtopics, and each subtopic is then broken down into keywords that cover the entire scientific aspect of that subtopic.

**Figure 5:**
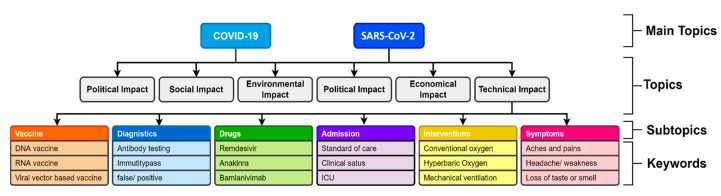
Sample list of keywords for SARS-CoV-2 and COVID-19 disease.

For instance, the KnowVID-19 system facilitates medical researchers in efficiently navigating through subtopics and related keywords, allowing them to quickly access specific information. This is achieved by storing structured keyword lists in a database, particularly for subtopics associated with Figure 5. The system enables smooth transitions between subtopics and relevant keywords.

The following guidelines define the structure of topics, subtopics, keywords, and subkeywords, as outlined by the medical researchers:

- Topics: Broad categories related to a domain, such as Vaccination, Pandemics, Diagnostics, and Drugs (e.g., Vaccination in Figure 5).
- Subtopics: Specific subdomains within a topic, for instance, under Vaccination, subtopics include RNA vaccines, DNA vaccines, and recombinant vaccines.
- Keywords: Detailed discussions within subtopics, such as the production, delivery, and clinical trials of RNA vaccines (e.g., Vaccination > RNA Vaccine > Production, delivery, clinical trial).
- Subkeywords: Further scientific breakdowns of the keywords, providing a granular discussion, such as clinical trial phases (e.g., Vaccination > RNA Vaccine > Clinical Trial > Phase I, Phase II, Phase III).

#### 2.2.5. Automatic Text Classification (ML Based)/Keywords Generation

**Figure 6:**
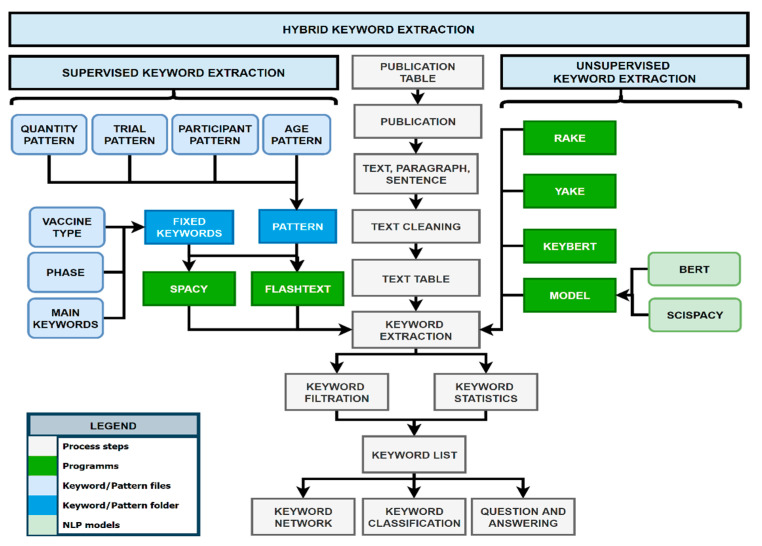
Diagram of keyword extraction process, showing the process of extracting the keyword from text, paragraph, and sentence.

The KnowVID-19 system uses a hybrid keyword extraction approach (see Figure 6) that combines machine learning and NLP techniques to identify relevant keywords from the collected data. This AI-powered approach enables the system to extract keywords and provide a comprehensive knowledge base for researchers and medical professionals.

A hybrid keyword extraction technique is employed, combining both supervised and unsupervised approaches. In the supervised method, predefined patterns and rules are used to identify keywords. In the unsupervised method, statistical and semantic-based algorithms are applied to automatically discover keywords from text.

The system employs NLP to automatically recognize named entities (keywords) in unprocessed text and categorize them into predetermined topics or fixed categories. A dictionary of terms/pattern or rules are constructed in a rule-based named entity recognition (NER) system based on the current knowledge base or vocabulary. Following that, these dictionary terms are tagged in the text using a string to exact match or a variant term that matches the established pattern [8,9,10]. In this case, multiple dictionaries containing different patterns and keywords from different topics and subtopics were created for the KnowVID-19, for example:

- Trial Pattern: Publication trials, like clinical trials or randomized clinical trials.
- Quantity Pattern: Quantities used for different scientific processes, like vaccination.
- Participant Pattern: Number of participants participating in biomedical research.
- Age Pattern: Age range of participants participating in biomedical research.
- Main Keywords: Important keywords and topics, like vaccination.
- Vaccination Type: Vaccine types, like mRNA vaccine (mRNA-1273).
- Phase Keywords: Phases describe the state of the vaccination or study (phase 1).

As shown in Figure 6, some unsupervised keyword extraction algorithms, such as RAKE (https://github.com/u-prashant/RAKE, accessed on 3 November 2024) (Rapid Automatic Keyword Extraction), Yake (Yet Another Keyword Extractor), and KeyBERT (https://github.com/MaartenGr/KeyBERT, accessed on 3 November 2024) (keyword extraction using BERT (Bidirectional Encoder Representations from Transformers)), were utilized to generate keywords and phrases automatically from the publications. For more information on these algorithms, please refer to [11,12,13].

At the end of the keyword generation process, a list of several keywords is then filtered out based on the importance and the statistics of that keyword. The following filtering criteria are implemented in addition:

- duplicated or similar keywords (example: randomized trials/randomized trials).
- same keywords with different description (example: controlled clinical trial/control clinical trial).
- meaningless or technical and scientific terms (example, yoga trial/young trial).
- grammar errors or unwanted symbols in the extracted keywords.
- total number of keywords in the text, paragraph, or document.
- importance of the keyword related to the content of the document.

It is noteworthy to mention that the tools used for keyword generation are carefully selected for their effectiveness in extracting contextually relevant keywords, which is crucial to building a comprehensive and searchable knowledge base within KnowVID-19. Each model brings distinct strengths to the extraction process. Together, these models allow the system to extract keywords with both speed and accuracy, significantly enhancing the user experience by reducing the time and effort required to locate specific topics and concepts within the literature. By combining these models, KnowVID-19 achieves a robust and multi-faceted approach to keyword extraction. RAKE and YAKE (https://github.com/LIAAD/yake, accessed on 3 November 2024) provide fast, frequency-based insights into important terms, while KeyBERT adds a semantic layer, ensuring that the extracted keywords are contextually meaningful within the biomedical field. This approach enhances the precision and relevance of information extraction, ensuring that KnowVID-19 presents users with terms and entities that are central to each document’s content and useful for exploring complex relationships within the literature.

#### 2.2.6. Text Classification Based on Term Frequency and Document Frequency

**Figure 7:**
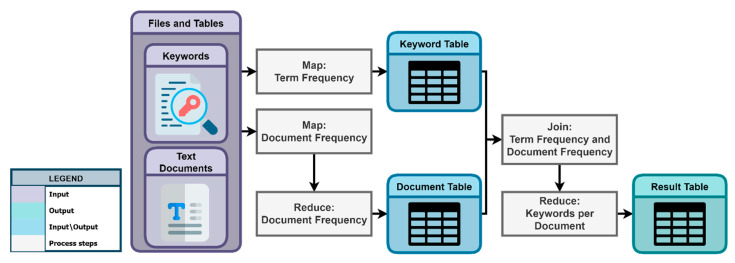
Keyword and text classification process.

After the keyword generation process, KnowVID-19 employs the Term Frequency (TF) and Document Frequency (DF) methods to finalize the text classification process. Figure 7 depicts the step-by-step process of employing these techniques on the given list of keywords and the text documents crawled and stored by the crawler.

Similarly, Table 1 provides a quick summary of the results of generated keywords, outlining their analysis through Document Frequency (A) and Term Frequency (B) methodologies. It presents an analysis of extracted keywords within the context of various topics, encompassing Trial type, Age group, and Dose. Subsection (A) of Table 1 comprises distinct tables delineating topics and their associated keywords, along with the corresponding count of publications featuring each keyword. Furthermore, Subsection (B) displays a detailed breakdown of the keywords and topics for a single publication, contrasting the number of topics and keywords across all publications.

Here, the classification of publications into different topics and subtopics based on the frequency of keywords and topics (Table 1A), as well as the prevalence of a given keyword within the document is executed via Inverse Document Frequency (IDF). This technique is conventionally employed to identify words that are frequent within an individual document yet possess uniqueness across all documents. For instance, an examination reveals a minimum of 2050 documents featuring the keyword “clinical trial” within the “Trial type” topic.

Conversely, the second table (Table 1B) is utilized to ascertain the TF through various methodologies. Preceding the subsequent analysis, irrelevant words pertaining to the document or publication are eliminated, as exemplified by the removal of the publication with ID 7591699. Additionally, terms such as “human clinical trial” that appear infrequently within the topic “TRIAL_TYPE” (less than 3 times) are excluded. Leveraging Document Frequency allows for the identification and elimination of words with low predictive power, enhancing the precision of subsequent analytical tasks. The frequency of a term within a document serves as a determinant of its significance within the overarching document or topic.

### 2.3. Interface

The KnowVID-19 system provides a user-friendly interface for exploring and utilizing the generated COVID-19 knowledge graph. The interface consists of three main components: network generation, visualization, and application.

#### 2.3.1. Network Generation

**Figure 8:**
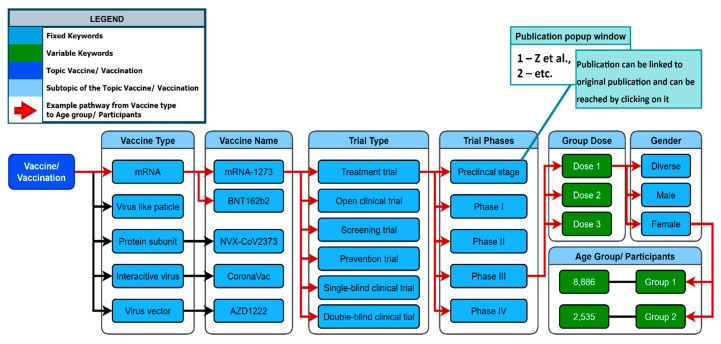
Keyword network, representing the topic of vaccination and its relation to other keywords (see further examples in Supplementary Figure S1).

The network generation process is used to visualize the generated keywords for multiple given topics and subtopics. To create a network, the topic that needs to be explored must be first defined (e.g., Vaccine). Identifying all subtopics related to the topic is the next step of the network generation process. The subtopics of the topic “Vaccine” may include “Vaccine Type”, “Vaccine Name”, and “Trial Type” (see Figure 8). After these topics have been identified, they are ranked by priority on a list.

The KnowVID-19 system used a NetworkX (https://networkx.org/) Python library to generate the network. The algorithm works to find out the most relevant words that are used in the text or publication, hence making it easier for network generation [14]. The NetworkX library is used for graph theory and networks creation. Leveraging the library’s sophisticated high-level interface, the process encompasses the proficient creation, manipulation, and in-depth analysis of diverse networks.

There are two major types of nodes in the network: fixed keywords and variable keywords (see Figure 8). Fixed keywords are nodes that have a specific meaning or relevance for the query or the analysis, e.g., mRNA vaccination. COVID-19, mRNA-1273, and BNT162b2 are examples of fixed keywords. In the variable keywords, the values are from the given paper that must be extracted and filled in. They can change or vary based on the paper given. Dose 1 (100 mg) and Age Group 1 (10 to 25 years) are examples of variable keywords.

#### 2.3.2. Visualization

KnowVID-19 uses Cytoscape software (https://cytoscape.org/) platform to visualize the keywords as a network. The Cytoscape program provides a basic set of features for integrating, analyzing, and visualizing keywords network data. The software also includes apps for network analysis, formatting, scripting, and database connection [15]. A subprogram of Cytoscape is Cytoscape.js, which is an open-source library for network graph visualization written in JavaScript. The Cytoscape.js library makes it easy to display and manipulate rich, interactive network graphs [16].

#### 2.3.3. Application

A web application was developed and trialed to facilitate exploration and use of the generated COVID-19 Knowledge Graph. For creating a responsive website, the bootstrap style web frameworks were used to build the front-end of the application. A python-based micro framework called “Flask” was used to implement the backend of the software. Flask offers all the basic features of a web application. Due to Flask’s templating engine (JinJa2—Jinja Template Engine 2), the framework also allowed for the creation of the entire website. This framework also offers many functionalities to develop a full-fledged REST (Representational State Transfer) API (Application Programming Interface) [17,18].

## 3. Results

The KnowVID-19 system was tested with various keywords related to COVID-19, and the results are presented below.

### 3.1. Search Results

**Figure 9:**
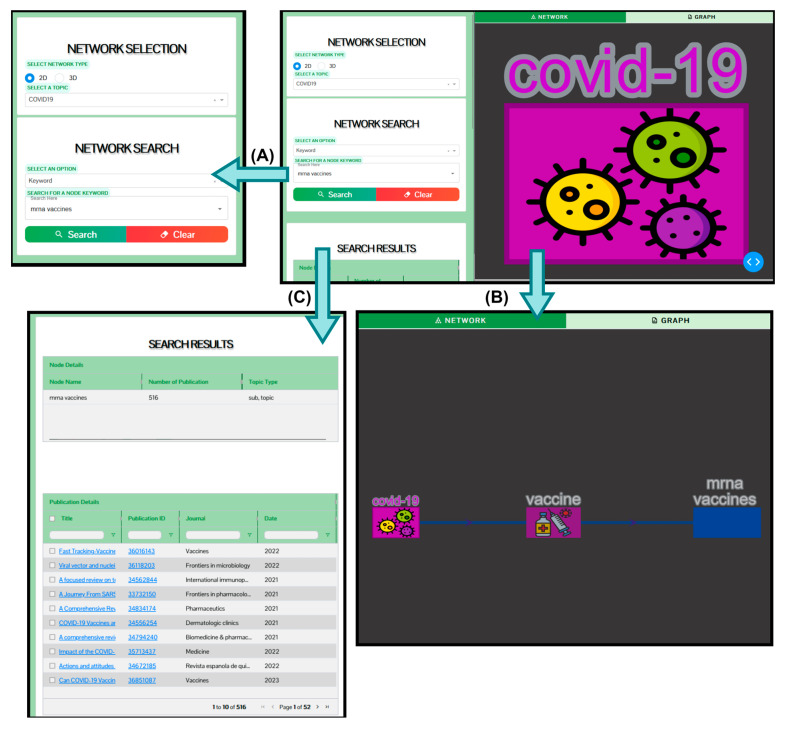
KnowVID-19 web-/graphic-interface showing the keyword network and a search bar. (**A**) search result of the keyword mrna vaccines, (**B**) expanded keyword network, and (**C**) search results of the keyword mrna vaccines in a detailed table format.

Figure 9 shows the search results for the keyword ‘mRNA-1273’ on the KnowVID-19 website. The right panel of Figure 9 displays a segment of the main network of KnowVID-19, which contains two nodes: COVID-19 and vaccination. The ‘COVID-19’ keyword is the central node, and the ‘vaccination’ keyword is linked to the central node. This representation can be expanded with other COVID-19- and long COVID-related main topics (see Section 2.2.3 for more information). Up to seven comma-separated keywords can be given in the search bar. Underneath the search bar appears the search results, which are publications containing the given keyword in order of frequency of the keyword.

The search results are visualized in a table format, showing the title of the publication and the URLs (Uniform Resource Locator) of the PubMed website (abstract only) and the original published publication website (full text or abstract only). In addition, Figure 9C shows the detailed version of the output search result table. The table not only shows the publication title and the URLs, but it also shows the authors, year of publication, and the journal in which the publication came from.

### 3.2. Filtering and Sorting Mechanisms

Figure 9C presents a comprehensive view of the output search results table, offering detailed information for each publication. Beyond simply listing the publication titles and URLs, the table also includes the authors, year of publication, and the journal where the publication appeared. Users can customize and refine the search results through several filtering options. The search bar allows users to quickly retrieve publications based on title, author, year of publication, keywords, or journal type. Additionally, the number of publications displayed can be reduced for more focused viewing. Filters can also be applied to narrow results by publication year or to organize authors alphabetically or by a specific name. For further customization, users can modify the table’s content by selecting which columns to display, ensuring that only the necessary information is visualized for better readability and analysis.

These filtering mechanisms offer significant benefits for users working with large sets of search results. For instance, if a user is looking for a specific article or a subset of publications within a certain date range, the ability to filter numerically by year allows for quicker identification of relevant content. Likewise, the option to sort and search by author helps users locate works from specific researchers or collaborators. The customization of visible columns further enhances user control, enabling more efficient navigation by hiding unnecessary data and presenting only essential information. This flexibility streamlines the process of reviewing academic publications, improving both productivity and user experience.

### 3.3. Network Visualization

As a response to the input data introduced in the search field on the KnowVID-19 website (Figure 9B), the visualized network subgraph focuses on the connection between COVID-19 and vaccination, and expands with new vaccination-related topics, represented as nodes. In general, the visualized network subgraph is generated with Cytoscape using all the keywords that relate to the input keyword(s). The KnowVID-19 website represents the keywords as nodes of a network graph. The edges represent the connection with other related keywords.

The keyword network representation facilitates locating and extracting the information more easily and quickly (see Figure 9B). The generated network has 485 nodes and 881 links between each node, where each node contains one to four words, and each node contains a list of publication IDs as an attribute. Every link connects multiple nodes together, and each connection shows that the words appear in multiple documents.

By pressing on a certain node, another network subgraph will appear with nodes that represent related keywords and directed lines are drawn from that node to all relating nodes. KnowVID-19 analyzes the existing search results and chooses keywords based on them. A graph, especially one that depicts a network of data, introduces the idea of data in terms of connections.

## 4. Discussion

The KnowVID-19 system has demonstrated its effectiveness in extracting targeted COVID-19 information and providing a comprehensive knowledge graph for researchers and medical professionals. In this section, we will discuss the evaluation of KnowVID-19 vis-à-vis established paradigms, its functionalities, limitations, and proposed improvements.

### 4.1. Functionalities

The KnowVID-19 system not only offers a web application to make the material available to the research community, but it also provides standard formats for graphs and networks that can be shared with other researchers. It is also possible to combine high-quality manual curated knowledge with scalable text mining approaches, such as NLP, to extract knowledge from COVID-19 and long COVID literature, allowing for the construction of knowledge graphs or networks based on entity co-occurrence or relation extraction.

Further important functionalities of KnowVID-19 are:

- It offers a quick and comprehensive KBS of COVID-19 medical information and helps keep track of the latest research and findings on COVID-19.
- The COVID-19 dataset is represented in a detailed table format that includes publication title, author’s names and their institutions, paper sections, and annotated references.
- It allows the users to easily search for specific information related to COVID-19 without going to the manuscript.
- The system can be used to generate specialized queries and search results.
- It can extract keywords from publication, articles, and biomedical other sources and analyze the most frequently used keywords by the sources and publications.
- A real-time monitoring system for publication, articles, and other sources
- By combining semantics and grammar, the dataset, and the context of the words, information can be extracted and analyzed.
- It saves storage and computing resources by reducing the word length instead of the whole text.

### 4.2. Limitations

While the KnowVID-19 system has demonstrated promising results in extracting targeted COVID-19 information, there are several AI-related challenges and limitations that need to be addressed. For example, the system requires large amounts of training data to improve its accuracy, and there is a risk of bias in the AI models used for keyword extraction. Despite the efficient search of data using a web crawler, some challenges remain e.g., numerous websites having anti-crawling restrictions, making it difficult to acquire the required data information. One example of such a restriction is with robot.txt. Robots.txt is a file used by websites to inform search bots whether the site should be crawled. In addition, some sites simply restrict crawling, which means that the KnowVID-19 system avoids those sites. For Example, KnowVID-19 can web crawl simple websites, like PubMed and PMC, but when it encounters a more complex website, with complex restrictions, they may become uncrawlable. An example of such websites is those that restrict the downloading of a PDF file, needed for further analysis, due to being locked behind a paywall or subscription, or the website does not give access to the content of the publication.

On the other hand, while KnowVID-19 currently operates with a relatively small dataset, it is anticipated that this dataset will grow significantly in the near future. As the system continues to gather more data from an increasing number of sources, the scope and size of the dataset will inevitably expand. This growth reflects the rapid pace at which new research and publications, especially in the context of COVID-19, are being produced. As such, while the current dataset is manageable, scaling to accommodate a larger corpus of information will introduce new challenges, particularly in terms of data processing and system performance.

The web crawling task, which is a core component of how KnowVID-19 collects and updates its dataset, will become increasingly complex as the volume of data grows. Web crawling involves systematically searching and retrieving data from various sources across the internet, and while it functions efficiently at smaller scales, handling a much larger dataset could prove to be highly resource-intensive. As the number of target websites and research repositories increases, the time and computational resources required for crawling, indexing, and updating data will rise exponentially. In a large-scale operation, the system will need to handle not only a greater volume of data but also more frequent updates to ensure the most current research is available. This could lead to significant increases in processing time, bandwidth usage, and storage requirements, making the task more time-consuming and complex.

Moreover, the complexity of web crawling at scale also introduces additional technical challenges, such as optimizing the crawling algorithms to prioritize the most relevant or recently updated sources, managing data deduplication, and ensuring the accuracy and completeness of the crawled data. As the dataset grows, it will be crucial to implement advanced strategies for data management and processing, including distributed crawling techniques, intelligent prioritization systems, and efficient data storage solutions. Addressing these challenges will be essential to ensure that KnowVID-19 continues to function smoothly and efficiently as it scales to handle an ever-growing body of research data.

#### Proposed Improvements

Circumventing web crawling restrictions, such as those imposed by robots.txt files or limitations on PDF downloads, is widely considered unethical and can undermine trust within the research and scientific communities. However, there are ethical and effective alternatives for acquiring data that could significantly enhance the visibility, credibility, and functionality of the KnowVID-19 system.

One of the most promising approaches is the integration of third-party APIs. These APIs offer controlled access to high-quality, curated, and frequently updated datasets while ensuring full compliance with the terms of service set by the data providers. This approach not only provides a reliable and legal means of accessing data but also guarantees that the KnowVID-19 system remains aligned with best practices in data use and management.

Additionally, it is critical to respect the web crawling guidelines set by websites. This includes adhering to the restrictions specified in robots.txt files, abiding by rate limits to prevent server overload, and ensuring responsible data collection practices. Respecting these guidelines not only helps in maintaining ethical standards but also fosters positive relationships with data providers, ensuring long-term access to relevant information.

Moreover, from the data limitations point-of-view, the KnowVID-19 system has been engineered with a high degree of flexibility, allowing it to collect and aggregate data from a wide range of sources, both peer-reviewed and prepeer-reviewed. This adaptability is a key feature, as it enables the system to tap into various scholarly databases and repositories that host scientific literature. For instance, KnowVID-19 can gather data from platforms such as Google Scholar, which covers a broad spectrum of academic publications, as well as preprint servers like medRxiv.org, bioRxiv.org, and arXiv.org. These repositories are critical for COVID-19 research, particularly in the early stages of the pandemic when timely access to prepeer-reviewed data was crucial. The system’s ability to seamlessly integrate information from such diverse sources ensures that users have access to the latest research, enabling a comprehensive view of the evolving scientific landscape.

In addition to its flexibility in data sources, the KnowVID-19 system has demonstrated reliability during scaling attempts, which is an essential consideration given the exponential growth of COVID-19 research. As new data continues to emerge, the system’s ability to handle significantly larger datasets has been thoroughly tested, proving its robustness and scalability. Whether managing a modest collection of research articles or processing vast amounts of data, KnowVID-19 has shown that it can efficiently accommodate the growing influx of COVID-19 literature. This scalability ensures that the system remains relevant and functional as research continues to expand, providing a reliable tool for researchers, clinicians, and policymakers who rely on up-to-date information to make informed decisions in the fight against the pandemic.

By addressing these limitations and adopting these ethical data acquisition strategies, the KnowVID-19 system can grow into a more robust, reliable, and respected tool for extracting targeted COVID-19 information. This would ultimately facilitate more efficient and accurate research, thereby contributing to the advancement of knowledge and scientific progress within the medical and research communities.

### 4.3. Example Scenarios Demonstrating the Capacity of KnowVID-19

Scenario 1: Quick Detection of COVID-19 Vaccine Effectiveness Research

Context: With a particular focus on mRNA vaccines like Pfizer-BioNTech and Moderna, a research team has been charged with swiftly locating all pertinent papers on the effectiveness of COVID-19 vaccines.

How KnowVID-19 could help for this scenario:

- Interactive Node-Based Navigation: Researchers may dive down into subnodes like “Pfizer-BioNTech” and “Moderna” by clicking on nodes labeled “mRNA Vaccines” using KnowVID-19’s online interface. This feature arranges data hierarchically, making it easier to browse through large datasets.
- Instant Access to papers: By selecting one of these nodes, you may obtain a well selected list of papers from PubMed instantly. Researchers may now save a great deal of time and effort by not having to manually sort through thousands of publications.
- Focused Information: The system groups pertinent information into categories like “symptoms”, and so on.
- Keyword Search and Visualization: To locate certain information in the dataset, researchers can make use of the keyword search tool.
- Trial Quantities: Researchers can also see the quantities of each component used in the vaccine trials, providing detailed insights into dosage usage and formulation specifics.
- Age Range of Participants: The system includes data on the age range of participants in the vaccine trials, allowing researchers to understand the demographics of the study groups.

Comparison: KnowVID-19 offers targeted, node-based navigation and precise classification, whereas CovidPubGraph offers a wide overview and knowledge graph of COVID-19 publications based on CORD-19 data. Although KG-COVID-19 offers a large dataset as well, it does not give the same degree of interactive, user-friendly navigation or focused data extraction. Comparing the broader and less interactive frameworks of CovidPubGraph and KG-COVID-19 to KnowVID-19, one can see a substantial improvement in research efficiency and accuracy due to the latter’s fewer interactive features and fast access to precisely classified data.

Scenario 2: Examining Prolonged COVID-19 Symptoms and Trials Background: A group of medical experts is looking at the variety of symptoms linked to long COVID and the studies where these problems were recorded.

How KnowVID-19 could help for this scenario:

- Symptom and Trial Nodes: When a user clicks on a node that represents a symptom, such as “fatigue”, “brain fog”, or “shortness of breath”, linked trials are displayed right away. This methodical technique facilitates finding the needed information quickly.
- Detailed Visualizations: By using network graphs to show the relationships between trials and symptoms, it is simpler to determine which trials mentioned particular symptoms. These graphic aids assist in deciphering intricate linkages and spotting trends in the frequency of symptoms among several trials.
- Keyword Search and Visualization: Using a keyword, medical practitioners can look for certain symptoms or trials.

Comparison: In contrast, KG-COVID-19 supports complex queries, it lacks the intuitive visual representation of symptom and trial relationships. CovidPubGraph offers extensive linked data and NLP results, but it lacks the detailed visualizations and interactive symptom-trial mapping available in KnowVID-19. KnowVID-19 is a more potent tool for examining long COVID symptoms and trials because of its capacity to depict intricate data correlations and provide focused, interactive data retrieval.

### 4.4. Evaluation vis-à-vis Established Paradigms

To assess the KnowVid-19 system against established benchmarks, we compared it with two existing knowledge graph systems (Table 2) designed for managing COVID-19 data and facilitating research: CovidPubGraph [19] and KG-COVID-19 [20]. KnowVID-19 stands out due to its user-focused design, providing customized information retrieval, strong data visualization using Cytoscape, and accurate information extraction. It is highly adaptable and dynamic, integrating multiple Python libraries for processing data.

In contrast, CovidPubGraph prioritizes wide dataset integration with a focus on named entity recognition and linking but does not specifically aim at visual data presentation. KG-COVID-19 is designed to be flexible by enabling the creation of customizable knowledge graphs for various applications. It gives priority to complex queries and hypothesis-based visualization while placing less emphasis on direct visual representation.

KnowVID-19 sets itself apart from tools like CovidPubGraph and KG-COVID-19 by focusing on tailored information retrieval, adaptability, and enriched data visualization. Unlike other tools that provide broad overviews, KnowVID-19 delivers precise, contextually relevant search results, making it highly user-centric. It also includes unique data elements, such as participant numbers, dosage amounts, and age groups, which traditional knowledge graphs often overlook.

In conclusion, KnowVID-19 demonstrates superior performance compared to other knowledge graph systems in COVID-19 research owing to its customized information retrieval, exceptional adaptability, sophisticated data processing, and inventive visualization methods. It establishes a novel benchmark for effectiveness and understanding, offering potential to transform not only pandemic-related investigations but also wider scientific inquiries.

## 5. Conclusions and Future Work

The KnowVID-19 system demonstrates the potential of machine learning and artificial intelligence in improving the accuracy and efficiency of COVID-19 research. By leveraging AI techniques, researchers and medical professionals can quickly and accurately extract targeted information from online medical repositories, enabling faster and more effective decision-making.

The KnowVID-19 is a knowledge-based expert for searching scholarly information about medical and biological developments regarding COVID-19 and long COVID.

KnowVID-19 is capable of swiftly reading and comprehending biological research materials. The system’s AI powered NLP capabilities enable it to accurately extract relevant information from large volumes of text data, providing researchers and medical professionals with a comprehensive knowledge base. It can look for keywords in a text, extract information, and identify articles that are relevant to the keywords given. It can also answer queries regarding medical keywords, making it an excellent system for medical specialists with expert knowledge but who have limited time.

## 6. Future Plans

The forthcoming enhancements for the KnowVID-19 system involve strategic upgrades and improvements aimed at ensuring the provision of the latest information. The focus is on maximizing integration and fostering crosstalk with similar processes to enhance overall functionality. The upcoming updates are outlined below:

- Implementation of a feature to read figures and pictures for information extraction. This feature will enable the system to extract relevant information from visual data, such as images and graphs, which will further enhance its capabilities.
- Enhancement of data visualization capabilities to provide more comprehensive and user-friendly representations. This will enable researchers and medical professionals to better understand and interpret the extracted information.
- Integration of advanced ML algorithms to enhance the system’s predictive analytics and decision-making capabilities. This will enable the system to provide more accurate and reliable predictions and recommendations.
- Implementation of real-time collaboration features, facilitating seamless information sharing and collaborative analysis among users. This will enable researchers and medical professionals to work together more effectively and efficiently.
- Integrate fake news detection models to improve the quality and reliability of extracted information by filtering out misleading or unreliable content. This will enable the system to provide more accurate and reliable information.
- Exploration the use of large language models (LLMs). These models have the potential to improve both the accuracy and contextual understanding of extracted information, making them ideal for handling diverse and complex data sources.

By implementing these enhancements, the KnowVID-19 system will become an even more powerful tool for researchers and medical professionals, enabling them to quickly and accurately extract targeted information from online medical repositories and make more informed decisions.

## Figures and Tables

**Table 1:** Brief summary of the extracted keywords, outlining their analysis through Document Frequency (**A**) and Term Frequency (**B**) methodologies.

(A) Document Frequency						
Trial Type			Age Group			
Keywords	ID_List	Count	Keywords	ID_List		Count
clinical trial	[8830978, 7749639, et al.]	2050	18 to 55 years	[7472384, 8313090, et al.]		70
controlled trial	[7235585, 8382475, et al.]	1107	18 to 59 years	[7472384, 8630786, et al.]		39
randomized controlled trial	[8382475, 7460877, et al.]	674	18 to 65 years	[8423936, 8839300, et al.]		24
randomized clinical trial	[7235585, 7573513, et al.]	373	12 to 17 years	[8604800, 9350282, et al.]		22
randomized trial	[7573513, 7460877, et al.]	352	12 to 15 years	[8461570, 8711308, et al.]		21
placebo-controlled trial	[7885317, 8813065, et al.]	248	18 to 64 years	[8084611, 8585490, et al.]		20
controlled clinical trial	[7683586, 7527945, et al.]	196	65 to 85 years	[7706592, 8695521, et al.]		18
ongoing clinical trial	[7683586, 7906827, et al.]	133	18 to 60 years	[8014753, 7362821, et al.]		17
double-blind trial	[7527945, 8813065, et al.]	125	18 to 80 years	[8872486, 8871718, et al.]		17
open-label trial	[7445008, 7263255, et al.]	121	18 to 55 years	[8395838, 7821985, et al.]		13
**(B) Term Frequency**						
**Number of topics**			**Number of words per topic (one publication)**			
**ID**	**Number_Topic**	**Number_Keywords**	**ID**	**Topic**	**Keywords**	**Counts**
7591699	7	87	7591699	AGE	10 to 15 year	2
8704728	7	62	7591699	GENDER	men	2
7706592	7	61	7591699	GENDER	women	1
7824305	7	49	7591699	PARTCIPANTS	30,000 volunteer	3
9062866	7	46	7591699	QUANTITY_MG	100 ug	5
7990482	7	42	7591699	TRIAL_PHASE	phase 1	15
7583697	7	41	7591699	TRIAL_PHASE	phase 3	20
8482810	7	41	7591699	TRIAL_TYPE	clinical trial	35
9106357	7	41	7591699	TRIAL_TYPE	human clinical trial	3
9127699	7	36	7591699	VACCINE_NAMES	azd1222	18
8776284	7	35	7591699	VACCINE_NAMES	mrna-1273	3

**Table 2:** Overview of different systems for COVID-19 data management, focusing on their design, adaptability, visualization capabilities, and tools integration.

Feature	KnowVID-19	CovidPubGraph	KG-COVID-19
User-Centric Design	Tailored information retrieval for specific research needs.	Broad dataset integration without a focus on individual user queries.	Framework for producing customizable KGs for various COVID-19 applications.
Adaptability	Easily customizable to various scientific topics.	Structured for COVID-19 publications with emphasis on dataset interoperability.	Flexible framework for integrating biomedical data.
Integrated Tools	Leverages multiple Python libraries for robust processing.	Focuses on named entity recognition and linking.	Uses KGX for graph manipulation, and emphasizing data summarization.
Contextual Visualization	Visual representation of linkages between information.	Data linkage and interoperability without emphasis on visual context.	Supports hypothesis-based querying and visualization.
Precision in Information Extraction	Offers refined search outputs for highly specific datasets or insights.	Broad overview and linkage of publications, not tailored for specific queries.	Supports complex queries for drug repurposing and disease understanding.
Dynamic Adaptation	Capable of adapting to new information sources and formats.	Updated regularly but with less emphasis on dynamic content adaptation.	Regular updates incorporate new and updated data.
Data Visualization	Utilizes Cytoscape for intuitive network graphs.	Provides a comprehensive RDF knowledge graph without a specific focus on visual presentation.	Less emphasis on direct visual representation.
Data Sources	PubMed, PMC	CORD-19	Various biomedical sources, less specific.
Data Usage	Real-time data extraction and updating.	Regularly updated but less dynamic.	Supports regular updates, but less dynamic adaptation.
Date Extracted From	PubMed and PMC, up-to-date as of 2024	CORD-19, varies by update schedule.	Various sources, date varies by updates.
Focused Data Access	Immediate access to highly curated and categorized data.	Broad overview requiring more manual effort for specific data extraction.	Extensive data but less focused categorization.
Age Filter in the Network	Yes, allows categorizing by age range of trial participants.	No specific age range filter available.	No specific age range filter available.
Trial Phases Categorization	Yes, allows categorizing by trial phases (e.g., Phase III).	No specific trial phase categorization.	No specific trial phase categorization.
Quantity in Trials	Yes, provides details on quantities used in trials.	No detailed quantity information available.	No detailed quantity information available.

## Data Availability

Dataset available on request from the authors.

## Supplementary Materials

The following supporting information can be downloaded at: https://www.mdpi.com/article/10.3390/biom14111411/s1, Figure S1: Keyword Network for COVID-19-Related Topics.
