# Supplementary material for: KnowVID-19: A Knowledge-Based System to Extract Targeted COVID-19 Information from Online Medical Repositories
Source: Biomolecules. 2024 Nov 6;14(11):1411. doi: 10.3390/biom14111411 (PMC11592241; doi:10.3390/biom14111411)
Supplement: Supplementary file 1 [file biomolecules-14-01411-s001.zip › biomolecules-3174038-supplementary.pdf]

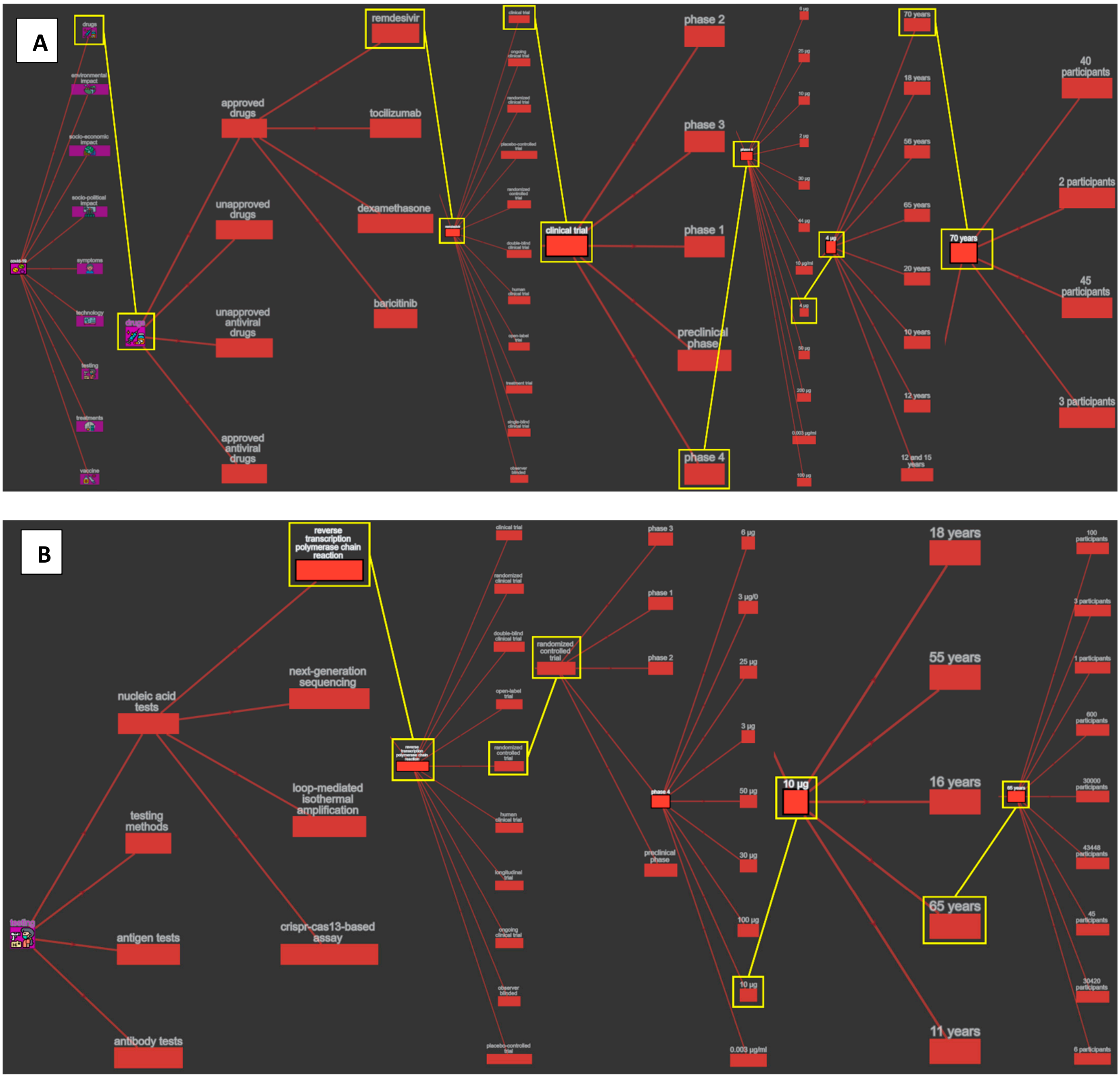

Supplementary Figure 1: Keyword Network for COVID-19-Related Topics

Keyword network, mapping the relationships between COVID-19-related topics and their associated keywords. **(A) Drugs:** This cluster highlights keywords associated with pharmacological treatments (drugs), including antiviral agents, and repurposed drugs. The connections illustrate how these terms are interlinked, reflecting shared research focus or clinical applications in the COVID-19 context. **(B) Testing:** This cluster focuses on keywords related to diagnostic methods. The network displays associations with key terms such as PCR, and NGS, emphasizing the breadth of diagnostic approaches used during the pandemic.

The network structure reflects the proximity of topics based on co-occurrence in the literature, allowing for an understanding of how these areas are interconnected within the broader COVID-19 research landscape. Each node is associated with its own set of publications, which are not displayed.
